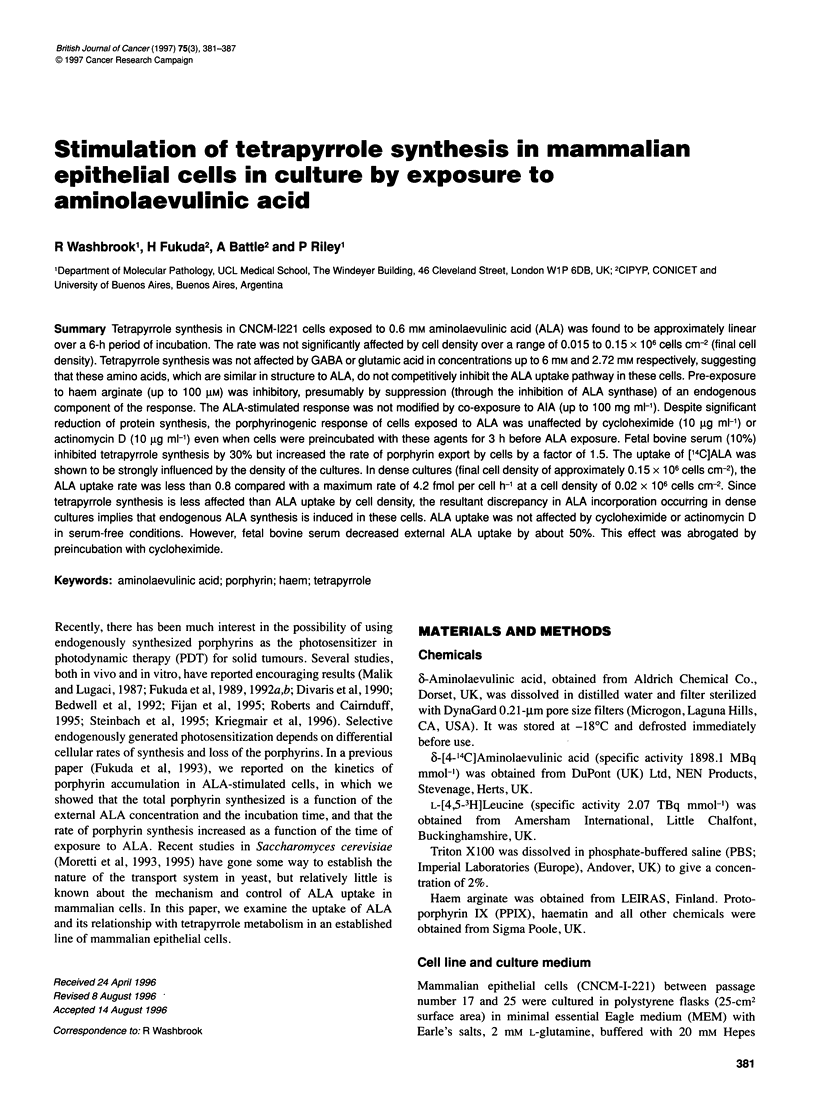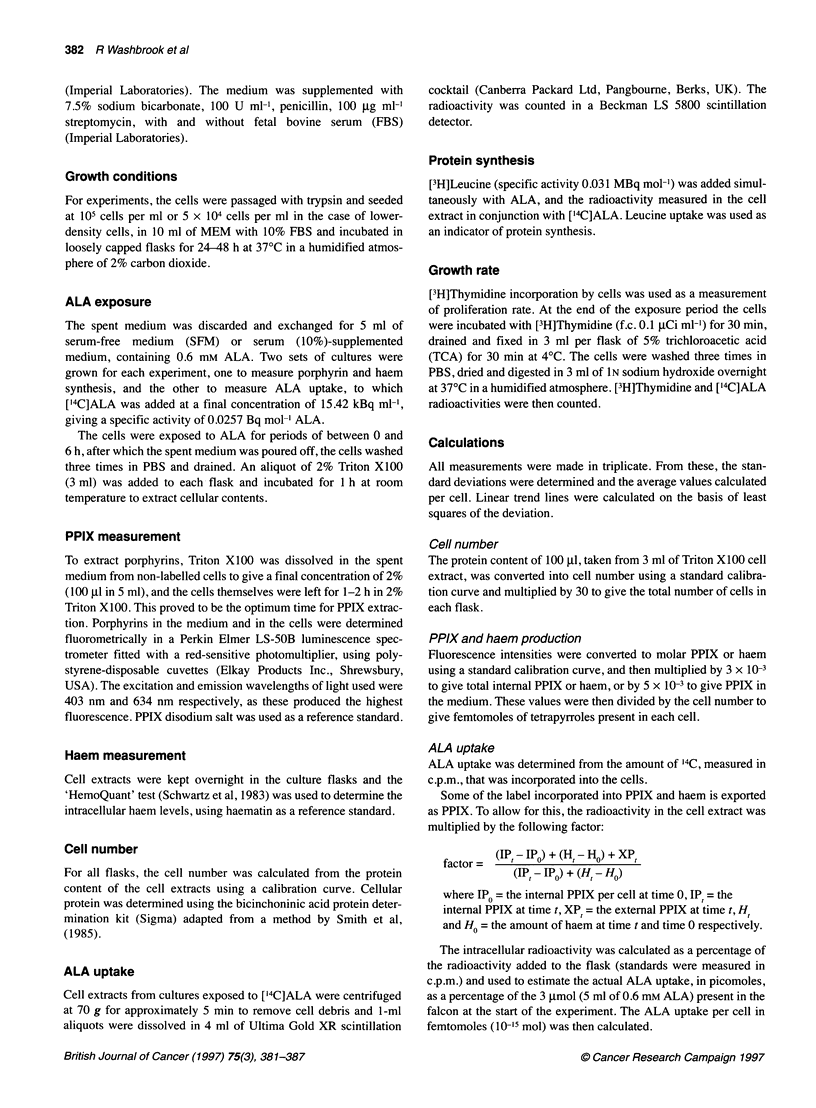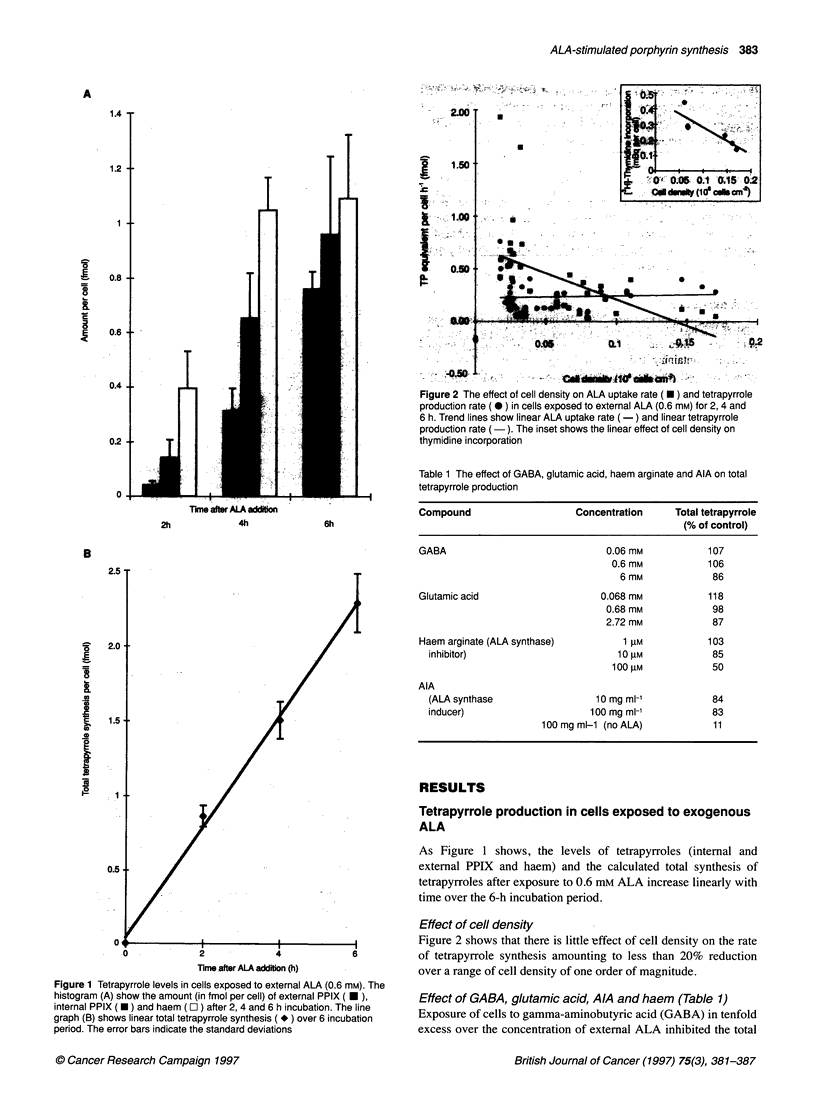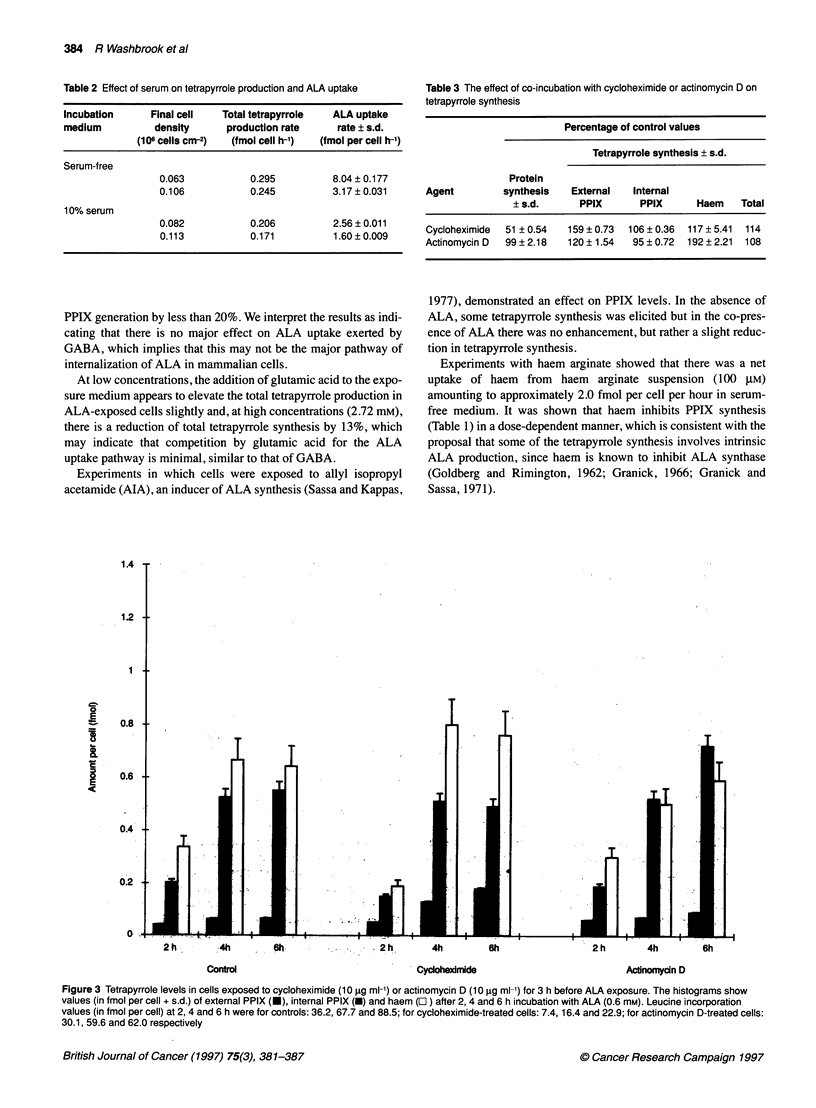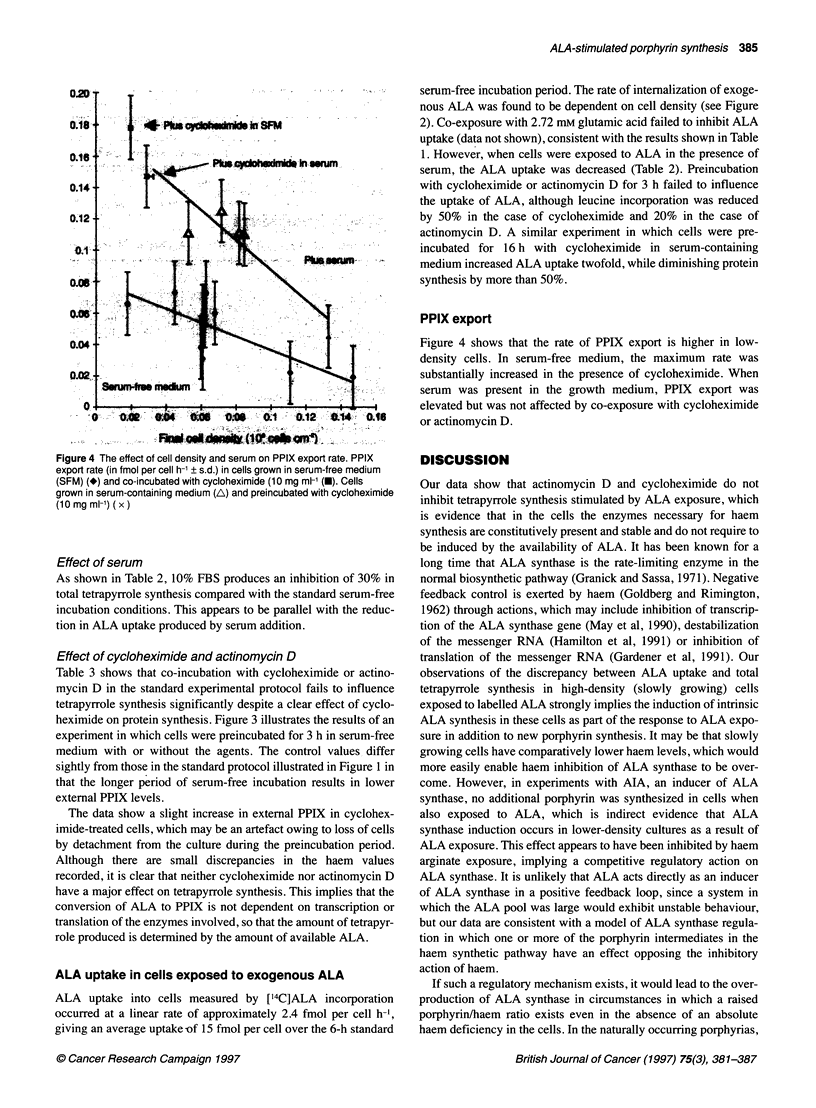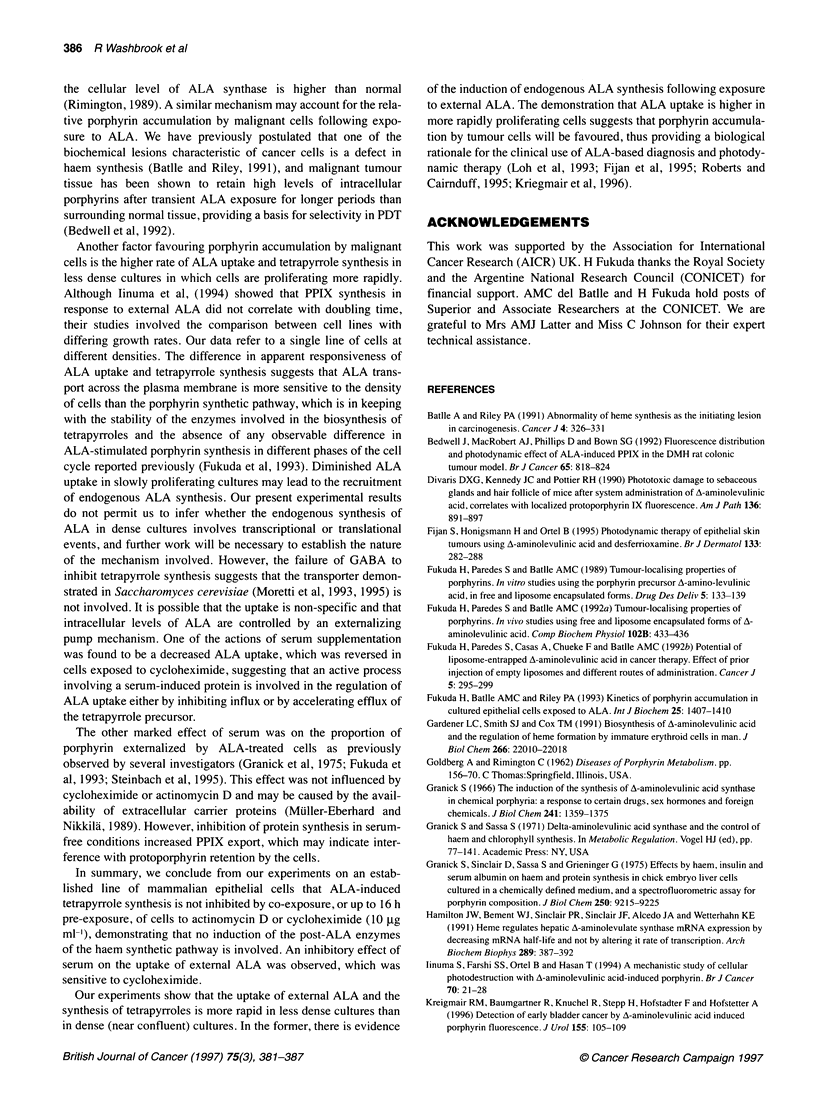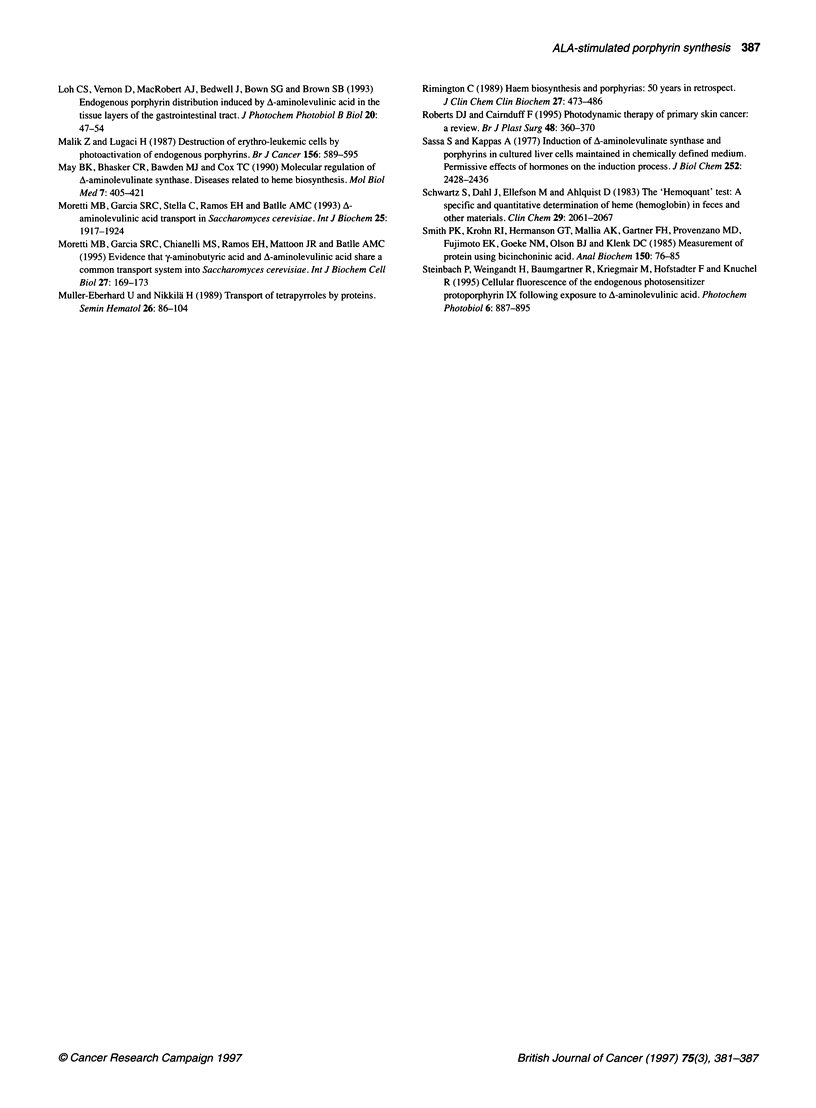# Stimulation of tetrapyrrole synthesis in mammalian epithelial cells in culture by exposure to aminolaevulinic acid.

**DOI:** 10.1038/bjc.1997.62

**Published:** 1997

**Authors:** R. Washbrook, H. Fukuda, A. Battle, P. Riley

**Affiliations:** Department of Molecular Pathology, UCL Medical School, London, UK.

## Abstract

Tetrapyrrole synthesis in CNCM-1221 cells exposed to 0.6 mM aminolaevulinic acid (ALA) was found to be approximately linear over a 6-h period of incubation. The rate was not significantly affected by cell density over a range of 0.015 to 0.15 x 10(6) cells cm(-2) (final cell density). Tetrapyrrole synthesis was not affected by GABA or glutamic acid in concentrations up to 6 mM and 2.72 mM respectively, suggesting that these amino acids, which are similar in structure to ALA, do not competitively inhibit the ALA uptake pathway in these cells. Pre-exposure to haem arginate (up to 100 microM) was inhibitory, presumably by suppression (through the inhibition of ALA synthase) of an endogenous component of the response. The ALA-stimulated response was not modified by co-exposure to AIA (up to 100 mg ml(-1)). Despite significant reduction of protein synthesis, the porphyrinogenic response of cells exposed to ALA was unaffected by cycloheximide (10 microg ml(-1)) or actinomycin D (10 microg ml(-1)) even when cells were preincubated with these agents for 3 h before ALA exposure. Fetal bovine serum (10%) inhibited tetrapyrrole synthesis by 30% but increased the rate of porphyrin export by cells by a factor of 1.5. The uptake of [14C]ALA was shown to be strongly influenced by the density of the cultures. In dense cultures (final cell density of approximately 0.15 x 10(6) cells cm(-2)), the ALA uptake rate was less than 0.8 compared with a maximum rate of 4.2 fmol per cell h(-1) at a cell density of 0.02 x 10(6) cells cm(-2). Since tetrapyrrole synthesis is less affected than ALA uptake by cell density, the resultant discrepancy in ALA incorporation occurring in dense cultures implies that endogenous ALA synthesis is induced in these cells. ALA uptake was not affected by cycloheximide or actinomycin D in serum-free conditions. However, fetal bovine serum decreased external ALA uptake by about 50%. This effect was abrogated by preincubation with cycloheximide.


British Journal of Cancer (1997) 75(3), 381–387
© 1997 Cancer Research Campaign

### Stimulation of tetrapyrrole synthesis in mammalian epithelial cells in culture by exposure to aminolaevulinic acid

**R Washbrook[1], H Fukuda[2], A Battle[2] and P Riley[1]**

[1]Department of Molecular Pathology, UCL Medical School, The Windeyer Building, 46 Cleveland Street, London W1P 6DB, UK; [2]CIPYP, CONICET and University of Buenos Aires, Buenos Aires, Argentina

**Summary** Tetrapyrrole synthesis in CNCM-I221 cells exposed to 0.6 mM aminolaevulinic acid (ALA) was found to be approximately linear over a 6-h period of incubation. The rate was not significantly affected by cell density over a range of 0.015 to $0.15 \times 10^6$ cells $cm^{-2}$ (final cell density). Tetrapyrrole synthesis was not affected by GABA or glutamic acid in concentrations up to 6 mM and 2.72 mM respectively, suggesting that these amino acids, which are similar in structure to ALA, do not competitively inhibit the ALA uptake pathway in these cells. Pre-exposure to haem arginate (up to 100 μM) was inhibitory, presumably by suppression (through the inhibition of ALA synthase) of an endogenous component of the response. The ALA-stimulated response was not modified by co-exposure to AIA (up to 100 mg $ml^{-1}$). Despite significant reduction of protein synthesis, the porphyrinogenic response of cells exposed to ALA was unaffected by cycloheximide (10 μg $ml^{-1}$) or actinomycin D (10 μg $ml^{-1}$) even when cells were preincubated with these agents for 3 h before ALA exposure. Fetal bovine serum (10%) inhibited tetrapyrrole synthesis by 30% but increased the rate of porphyrin export by cells by a factor of 1.5. The uptake of [$^{14}C$]ALA was shown to be strongly influenced by the density of the cultures. In dense cultures (final cell density of approximately $0.15 \times 10^6$ cells $cm^{-2}$), the ALA uptake rate was less than 0.8 compared with a maximum rate of 4.2 fmol per cell $h^{-1}$ at a cell density of $0.02 \times 10^6$ cells $cm^{-2}$. Since tetrapyrrole synthesis is less affected than ALA uptake by cell density, the resultant discrepancy in ALA incorporation occurring in dense cultures implies that endogenous ALA synthesis is induced in these cells. ALA uptake was not affected by cycloheximide or actinomycin D in serum-free conditions. However, fetal bovine serum decreased external ALA uptake by about 50%. This effect was abrogated by preincubation with cycloheximide.

**Keywords:** aminolaevulinic acid; porphyrin; haem; tetrapyrrole

Recently, there has been much interest in the possibility of using endogenously synthesized porphyrins as the photosensitizer in photodynamic therapy (PDT) for solid tumours. Several studies, both in vivo and in vitro, have reported encouraging results (Malik and Lugaci, 1987; Fukuda et al, 1989, 1992*a,b*; Divaris et al, 1990; Bedwell et al, 1992; Fijan et al, 1995; Roberts and Cairnduff, 1995; Steinbach et al, 1995; Kriegmair et al, 1996). Selective endogenously generated photosensitization depends on differential cellular rates of synthesis and loss of the porphyrins. In a previous paper (Fukuda et al, 1993), we reported on the kinetics of porphyrin accumulation in ALA-stimulated cells, in which we showed that the total porphyrin synthesized is a function of the external ALA concentration and the incubation time, and that the rate of porphyrin synthesis increased as a function of the time of exposure to ALA. Recent studies in *Saccharomyces cerevisiae* (Moretti et al, 1993, 1995) have gone some way to establish the nature of the transport system in yeast, but relatively little is known about the mechanism and control of ALA uptake in mammalian cells. In this paper, we examine the uptake of ALA and its relationship with tetrapyrrole metabolism in an established line of mammalian epithelial cells.

*Received 24 April 1996*
*Revised 8 August 1996*
*Accepted 14 August 1996*

*Correspondence to:* R Washbrook

### MATERIALS AND METHODS

### Chemicals

δ-Aminolaevulinic acid, obtained from Aldrich Chemical Co., Dorset, UK, was dissolved in distilled water and filter sterilized with DynaGard 0.21-μm pore size filters (Microgon, Laguna Hills, CA, USA). It was stored at −18°C and defrosted immediately before use.

δ-[4-$^{14}C$]Aminolaevulinic acid (specific activity 1898.1 MBq $mmol^{-1}$) was obtained from DuPont (UK) Ltd, NEN Products, Stevenage, Herts, UK.

L-[4,5-$^3H$]Leucine (specific activity 2.07 TBq $mmol^{-1}$) was obtained from Amersham International, Little Chalfont, Buckinghamshire, UK.

Triton X100 was dissolved in phosphate-buffered saline (PBS; Imperial Laboratories (Europe), Andover, UK) to give a concentration of 2%.

Haem arginate was obtained from LEIRAS, Finland. Protoporphyrin IX (PPIX), haematin and all other chemicals were obtained from Sigma Poole, UK.

### Cell line and culture medium

Mammalian epithelial cells (CNCM-I-221) between passage number 17 and 25 were cultured in polystyrene flasks (25-$cm^2$ surface area) in minimal essential Eagle medium (MEM) with Earle's salts, 2 mM L-glutamine, buffered with 20 mM Hepes

(Imperial Laboratories). The medium was supplemented with 7.5% sodium bicarbonate, 100 U ml$^{-1}$, penicillin, 100 µg ml$^{-1}$ streptomycin, with and without fetal bovine serum (FBS) (Imperial Laboratories).

### Growth conditions

For experiments, the cells were passaged with trypsin and seeded at $10^5$ cells per ml or $5 \times 10^4$ cells per ml in the case of lower-density cells, in 10 ml of MEM with 10% FBS and incubated in loosely capped flasks for 24–48 h at 37°C in a humidified atmosphere of 2% carbon dioxide.

### ALA exposure

The spent medium was discarded and exchanged for 5 ml of serum-free medium (SFM) or serum (10%)-supplemented medium, containing 0.6 mM ALA. Two sets of cultures were grown for each experiment, one to measure porphyrin and haem synthesis, and the other to measure ALA uptake, to which [$^{14}$C]ALA was added at a final concentration of 15.42 kBq ml$^{-1}$, giving a specific activity of 0.0257 Bq mol$^{-1}$ ALA.

The cells were exposed to ALA for periods of between 0 and 6 h, after which the spent medium was poured off, the cells washed three times in PBS and drained. An aliquot of 2% Triton X100 (3 ml) was added to each flask and incubated for 1 h at room temperature to extract cellular contents.

### PPIX measurement

To extract porphyrins, Triton X100 was dissolved in the spent medium from non-labelled cells to give a final concentration of 2% (100 µl in 5 ml), and the cells themselves were left for 1–2 h in 2% Triton X100. This proved to be the optimum time for PPIX extraction. Porphyrins in the medium and in the cells were determined fluorometrically in a Perkin Elmer LS-50B luminescence spectrometer fitted with a red-sensitive photomultiplier, using polystyrene-disposable cuvettes (Elkay Products Inc., Shrewsbury, USA). The excitation and emission wavelengths of light used were 403 nm and 634 nm respectively, as these produced the highest fluorescence. PPIX disodium salt was used as a reference standard.

### Haem measurement

Cell extracts were kept overnight in the culture flasks and the 'HemoQuant' test (Schwartz et al, 1983) was used to determine the intracellular haem levels, using haematin as a reference standard.

### Cell number

For all flasks, the cell number was calculated from the protein content of the cell extracts using a calibration curve. Cellular protein was determined using the bicinchoninic acid protein determination kit (Sigma) adapted from a method by Smith et al, (1985).

### ALA uptake

Cell extracts from cultures exposed to [$^{14}$C]ALA were centrifuged at 70 *g* for approximately 5 min to remove cell debris and 1-ml aliquots were dissolved in 4 ml of Ultima Gold XR scintillation cocktail (Canberra Packard Ltd, Pangbourne, Berks, UK). The radioactivity was counted in a Beckman LS 5800 scintillation detector.

### Protein synthesis

[$^3$H]Leucine (specific activity 0.031 MBq mol$^{-1}$) was added simultaneously with ALA, and the radioactivity measured in the cell extract in conjunction with [$^{14}$C]ALA. Leucine uptake was used as an indicator of protein synthesis.

### Growth rate

[$^3$H]Thymidine incorporation by cells was used as a measurement of proliferation rate. At the end of the exposure period the cells were incubated with [$^3$H]Thymidine (f.c. 0.1 µCi ml$^{-1}$) for 30 min, drained and fixed in 3 ml per flask of 5% trichloroacetic acid (TCA) for 30 min at 4°C. The cells were washed three times in PBS, dried and digested in 3 ml of 1N sodium hydroxide overnight at 37°C in a humidified atmosphere. [$^3$H]Thymidine and [$^{14}$C]ALA radioactivities were then counted.

### Calculations

All measurements were made in triplicate. From these, the standard deviations were determined and the average values calculated per cell. Linear trend lines were calculated on the basis of least squares of the deviation.

### *Cell number*

The protein content of 100 µl, taken from 3 ml of Triton X100 cell extract, was converted into cell number using a standard calibration curve and multiplied by 30 to give the total number of cells in each flask.

### *PPIX and haem production*

Fluorescence intensities were converted to molar PPIX or haem using a standard calibration curve, and then multiplied by $3 \times 10^{-3}$ to give total internal PPIX or haem, or by $5 \times 10^{-3}$ to give PPIX in the medium. These values were then divided by the cell number to give femtomoles of tetrapyrroles present in each cell.

### *ALA uptake*

ALA uptake was determined from the amount of $^{14}$C, measured in c.p.m., that was incorporated into the cells.

Some of the label incorporated into PPIX and haem is exported as PPIX. To allow for this, the radioactivity in the cell extract was multiplied by the following factor:

$$\text{factor} = \frac{(\mathrm{IP}_t - \mathrm{IP}_0) + (\mathrm{H}_t - \mathrm{H}_0) + \mathrm{XP}_t}{(\mathrm{IP}_t - \mathrm{IP}_0) + (H_t - H_0)}$$

where $\mathrm{IP}_0$ = the internal PPIX per cell at time 0, $\mathrm{IP}_t$ = the internal PPIX at time $t$, $\mathrm{XP}_t$ = the external PPIX at time $t$, $H_t$ and $H_0$ = the amount of haem at time $t$ and time 0 respectively.

The intracellular radioactivity was calculated as a percentage of the radioactivity added to the flask (standards were measured in c.p.m.) and used to estimate the actual ALA uptake, in picomoles, as a percentage of the 3 µmol (5 ml of 0.6 mM ALA) present in the falcon at the start of the experiment. The ALA uptake per cell in femtomoles ($10^{-15}$ mol) was then calculated.

© Cancer Research Campaign 1997

**Figure 1** Tetrapyrrole levels in cells exposed to external ALA (0.6 mM). The histogram (A) show the amount (in fmol per cell) of external PPIX ( ■ ), internal PPIX ( ■ ) and haem ( □ ) after 2, 4 and 6 h incubation. The line graph (B) shows linear total tetrapyrrole synthesis ( ◆ ) over 6 incubation period. The error bars indicate the standard deviations

**Figure 2** The effect of cell density on ALA uptake rate ( ■ ) and tetrapyrrole production rate ( ● ) in cells exposed to external ALA (0.6 mM) for 2, 4 and 6 h. Trend lines show linear ALA uptake rate ( — ) and linear tetrapyrrole production rate ( — ). The inset shows the linear effect of cell density on thymidine incorporation

Table 1 The effect of GABA, glutamic acid, haem arginate and AIA on total tetrapyrrole production

| Compound | Concentration | Total tetrapyrrole (% of control) |
|---|---|---|
| GABA | 0.06 mM | 107 |
| | 0.6 mM | 106 |
| | 6 mM | 86 |
| Glutamic acid | 0.068 mM | 118 |
| | 0.68 mM | 98 |
| | 2.72 mM | 87 |
| Haem arginate (ALA synthase) inhibitor) | 1 µM | 103 |
| | 10 µM | 85 |
| | 100 µM | 50 |
| AIA (ALA synthase inducer) | 10 mg ml$^{-1}$ | 84 |
| | 100 mg ml$^{-1}$ | 83 |
| | 100 mg ml–1 (no ALA) | 11 |

### RESULTS

### Tetrapyrrole production in cells exposed to exogenous ALA

As Figure 1 shows, the levels of tetrapyrroles (internal and external PPIX and haem) and the calculated total synthesis of tetrapyrroles after exposure to 0.6 mM ALA increase linearly with time over the 6-h incubation period.

### *Effect of cell density*

Figure 2 shows that there is little effect of cell density on the rate of tetrapyrrole synthesis amounting to less than 20% reduction over a range of cell density of one order of magnitude.

### *Effect of GABA, glutamic acid, AIA and haem (Table 1)*

Exposure of cells to gamma-aminobutyric acid (GABA) in tenfold excess over the concentration of external ALA inhibited the total

© Cancer Research Campaign 1997

**Table 2** Effect of serum on tetrapyrrole production and ALA uptake

| Incubation medium | Final cell density ($10^6$ cells $cm^{-2}$) | Total tetrapyrrole production rate (fmol cell $h^{-1}$) | ALA uptake rate ± s.d. (fmol per cell $h^{-1}$) |
|---|---|---|---|
| Serum-free | | | |
| | 0.063 | 0.295 | 8.04 ± 0.177 |
| | 0.106 | 0.245 | 3.17 ± 0.031 |
| 10% serum | | | |
| | 0.082 | 0.206 | 2.56 ± 0.011 |
| | 0.113 | 0.171 | 1.60 ± 0.009 |

**Table 3** The effect of co-incubation with cycloheximide or actinomycin D on tetrapyrrole synthesis

| | Percentage of control values | | | | |
|---|---|---|---|---|---|
| | | Tetrapyrrole synthesis ± s.d. | | | |
| Agent | Protein synthesis ± s.d. | External PPIX | Internal PPIX | Haem | Total |
| Cycloheximide | 51 ± 0.54 | 159 ± 0.73 | 106 ± 0.36 | 117 ± 5.41 | 114 |
| Actinomycin D | 99 ± 2.18 | 120 ± 1.54 | 95 ± 0.72 | 192 ± 2.21 | 108 |

PPIX generation by less than 20%. We interpret the results as indicating that there is no major effect on ALA uptake exerted by GABA, which implies that this may not be the major pathway of internalization of ALA in mammalian cells.

At low concentrations, the addition of glutamic acid to the exposure medium appears to elevate the total tetrapyrrole production in ALA-exposed cells slightly and, at high concentrations (2.72 mM), there is a reduction of total tetrapyrrole synthesis by 13%, which may indicate that competition by glutamic acid for the ALA uptake pathway is minimal, similar to that of GABA.

Experiments in which cells were exposed to allyl isopropyl acetamide (AIA), an inducer of ALA synthesis (Sassa and Kappas, 1977), demonstrated an effect on PPIX levels. In the absence of ALA, some tetrapyrrole synthesis was elicited but in the co-presence of ALA there was no enhancement, but rather a slight reduction in tetrapyrrole synthesis.

Experiments with haem arginate showed that there was a net uptake of haem from haem arginate suspension (100 μM) amounting to approximately 2.0 fmol per cell per hour in serum-free medium. It was shown that haem inhibits PPIX synthesis (Table 1) in a dose-dependent manner, which is consistent with the proposal that some of the tetrapyrrole synthesis involves intrinsic ALA production, since haem is known to inhibit ALA synthase (Goldberg and Rimington, 1962; Granick, 1966; Granick and Sassa, 1971).

**Figure 3** Tetrapyrrole levels in cells exposed to cycloheximide (10 μg $ml^{-1}$) or actinomycin D (10 μg $ml^{-1}$) for 3 h before ALA exposure. The histograms show values (in fmol per cell + s.d.) of external PPIX (■), internal PPIX (■) and haem (□) after 2, 4 and 6 h incubation with ALA (0.6 mM). Leucine incorporation values (in fmol per cell) at 2, 4 and 6 h were for controls: 36.2, 67.7 and 88.5; for cycloheximide-treated cells: 7.4, 16.4 and 22.9; for actinomycin D-treated cells: 30.1, 59.6 and 62.0 respectively

© Cancer Research Campaign 1997

**Figure 4** The effect of cell density and serum on PPIX export rate. PPIX export rate (in fmol per cell h$^{-1}$ ± s.d.) in cells grown in serum-free medium (SFM) (◆) and co-incubated with cycloheximide (10 mg ml$^{-1}$ (■). Cells grown in serum-containing medium (△) and preincubated with cycloheximide (10 mg ml$^{-1}$) ( × )

### *Effect of serum*

As shown in Table 2, 10% FBS produces an inhibition of 30% in total tetrapyrrole synthesis compared with the standard serum-free incubation conditions. This appears to be parallel with the reduction in ALA uptake produced by serum addition.

### *Effect of cycloheximide and actinomycin D*

Table 3 shows that co-incubation with cycloheximide or actinomycin D in the standard experimental protocol fails to influence tetrapyrrole synthesis significantly despite a clear effect of cycloheximide on protein synthesis. Figure 3 illustrates the results of an experiment in which cells were preincubated for 3 h in serum-free medium with or without the agents. The control values differ sightly from those in the standard protocol illustrated in Figure 1 in that the longer period of serum-free incubation results in lower external PPIX levels.

The data show a slight increase in external PPIX in cycloheximide-treated cells, which may be an artefact owing to loss of cells by detachment from the culture during the preincubation period. Although there are small discrepancies in the haem values recorded, it is clear that neither cycloheximide nor actinomycin D have a major effect on tetrapyrrole synthesis. This implies that the conversion of ALA to PPIX is not dependent on transcription or translation of the enzymes involved, so that the amount of tetrapyrrole produced is determined by the amount of available ALA.

### ALA uptake in cells exposed to exogenous ALA

ALA uptake into cells measured by [$^{14}$C]ALA incorporation occurred at a linear rate of approximately 2.4 fmol per cell h$^{-1}$, giving an average uptake of 15 fmol per cell over the 6-h standard serum-free incubation period. The rate of internalization of exogenous ALA was found to be dependent on cell density (see Figure 2). Co-exposure with 2.72 mM glutamic acid failed to inhibit ALA uptake (data not shown), consistent with the results shown in Table 1. However, when cells were exposed to ALA in the presence of serum, the ALA uptake was decreased (Table 2). Preincubation with cycloheximide or actinomycin D for 3 h failed to influence the uptake of ALA, although leucine incorporation was reduced by 50% in the case of cycloheximide and 20% in the case of actinomycin D. A similar experiment in which cells were preincubated for 16 h with cycloheximide in serum-containing medium increased ALA uptake twofold, while diminishing protein synthesis by more than 50%.

### PPIX export

Figure 4 shows that the rate of PPIX export is higher in low-density cells. In serum-free medium, the maximum rate was substantially increased in the presence of cycloheximide. When serum was present in the growth medium, PPIX export was elevated but was not affected by co-exposure with cycloheximide or actinomycin D.

### DISCUSSION

Our data show that actinomycin D and cycloheximide do not inhibit tetrapyrrole synthesis stimulated by ALA exposure, which is evidence that in the cells the enzymes necessary for haem synthesis are constitutively present and stable and do not require to be induced by the availability of ALA. It has been known for a long time that ALA synthase is the rate-limiting enzyme in the normal biosynthetic pathway (Granick and Sassa, 1971). Negative feedback control is exerted by haem (Goldberg and Rimington, 1962) through actions, which may include inhibition of transcription of the ALA synthase gene (May et al, 1990), destabilization of the messenger RNA (Hamilton et al, 1991) or inhibition of translation of the messenger RNA (Gardener et al, 1991). Our observations of the discrepancy between ALA uptake and total tetrapyrrole synthesis in high-density (slowly growing) cells exposed to labelled ALA strongly implies the induction of intrinsic ALA synthesis in these cells as part of the response to ALA exposure in addition to new porphyrin synthesis. It may be that slowly growing cells have comparatively lower haem levels, which would more easily enable haem inhibition of ALA synthase to be overcome. However, in experiments with AIA, an inducer of ALA synthase, no additional porphyrin was synthesized in cells when also exposed to ALA, which is indirect evidence that ALA synthase induction occurs in lower-density cultures as a result of ALA exposure. This effect appears to have been inhibited by haem arginate exposure, implying a competitive regulatory action on ALA synthase. It is unlikely that ALA acts directly as an inducer of ALA synthase in a positive feedback loop, since a system in which the ALA pool was large would exhibit unstable behaviour, but our data are consistent with a model of ALA synthase regulation in which one or more of the porphyrin intermediates in the haem synthetic pathway have an effect opposing the inhibitory action of haem.

If such a regulatory mechanism exists, it would lead to the overproduction of ALA synthase in circumstances in which a raised porphyrin/haem ratio exists even in the absence of an absolute haem deficiency in the cells. In the naturally occurring porphyrias,

© Cancer Research Campaign 1997 

the cellular level of ALA synthase is higher than normal (Rimington, 1989). A similar mechanism may account for the relative porphyrin accumulation by malignant cells following exposure to ALA. We have previously postulated that one of the biochemical lesions characteristic of cancer cells is a defect in haem synthesis (Batlle and Riley, 1991), and malignant tumour tissue has been shown to retain high levels of intracellular porphyrins after transient ALA exposure for longer periods than surrounding normal tissue, providing a basis for selectivity in PDT (Bedwell et al, 1992).

Another factor favouring porphyrin accumulation by malignant cells is the higher rate of ALA uptake and tetrapyrrole synthesis in less dense cultures in which cells are proliferating more rapidly. Although Iinuma et al, (1994) showed that PPIX synthesis in response to external ALA did not correlate with doubling time, their studies involved the comparison between cell lines with differing growth rates. Our data refer to a single line of cells at different densities. The difference in apparent responsiveness of ALA uptake and tetrapyrrole synthesis suggests that ALA transport across the plasma membrane is more sensitive to the density of cells than the porphyrin synthetic pathway, which is in keeping with the stability of the enzymes involved in the biosynthesis of tetrapyrroles and the absence of any observable difference in ALA-stimulated porphyrin synthesis in different phases of the cell cycle reported previously (Fukuda et al, 1993). Diminished ALA uptake in slowly proliferating cultures may lead to the recruitment of endogenous ALA synthesis. Our present experimental results do not permit us to infer whether the endogenous synthesis of ALA in dense cultures involves transcriptional or translational events, and further work will be necessary to establish the nature of the mechanism involved. However, the failure of GABA to inhibit tetrapyrrole synthesis suggests that the transporter demonstrated in *Saccharomyces cerevisiae* (Moretti et al, 1993, 1995) is not involved. It is possible that the uptake is non-specific and that intracellular levels of ALA are controlled by an externalizing pump mechanism. One of the actions of serum supplementation was found to be a decreased ALA uptake, which was reversed in cells exposed to cycloheximide, suggesting that an active process involving a serum-induced protein is involved in the regulation of ALA uptake either by inhibiting influx or by accelerating efflux of the tetrapyrrole precursor.

The other marked effect of serum was on the proportion of porphyrin externalized by ALA-treated cells as previously observed by several investigators (Granick et al, 1975; Fukuda et al, 1993; Steinbach et al, 1995). This effect was not influenced by cycloheximide or actinomycin D and may be caused by the availability of extracellular carrier proteins (Müller-Eberhard and Nikkilä, 1989). However, inhibition of protein synthesis in serum-free conditions increased PPIX export, which may indicate interference with protoporphyrin retention by the cells.

In summary, we conclude from our experiments on an established line of mammalian epithelial cells that ALA-induced tetrapyrrole synthesis is not inhibited by co-exposure, or up to 16 h pre-exposure, of cells to actinomycin D or cycloheximide (10 $\mu g\ ml^{-1}$), demonstrating that no induction of the post-ALA enzymes of the haem synthetic pathway is involved. An inhibitory effect of serum on the uptake of external ALA was observed, which was sensitive to cycloheximide.

Our experiments show that the uptake of external ALA and the synthesis of tetrapyrroles is more rapid in less dense cultures than in dense (near confluent) cultures. In the former, there is evidence of the induction of endogenous ALA synthesis following exposure to external ALA. The demonstration that ALA uptake is higher in more rapidly proliferating cells suggests that porphyrin accumulation by tumour cells will be favoured, thus providing a biological rationale for the clinical use of ALA-based diagnosis and photodynamic therapy (Loh et al, 1993; Fijan et al, 1995; Roberts and Cairnduff, 1995; Kriegmair et al, 1996).

### ACKNOWLEDGEMENTS

This work was supported by the Association for International Cancer Research (AICR) UK. H Fukuda thanks the Royal Society and the Argentine National Research Council (CONICET) for financial support. AMC del Battle and H Fukuda hold posts of Superior and Associate Researchers at the CONICET. We are grateful to Mrs AMJ Latter and Miss C Johnson for their expert technical assistance.

### REFERENCES

Batlle A and Riley PA (1991) Abnormality of heme synthesis as the initiating lesion in carcinogenesis. *Cancer J* **4**: 326–331

Bedwell J, MacRobert AJ, Phillips D and Bown SG (1992) Fluorescence distribution and photodynamic effect of ALA-induced PPIX in the DMH rat colonic tumour model. *Br J Cancer* **65**: 818–824

Divaris DXG, Kennedy JC and Pottier RH (1990) Phototoxic damage to sebaceous glands and hair follicle of mice after system administration of Δ-aminolevulinic acid, correlates with localized protoporphyrin IX fluorescence. *Am J Path* **136**: 891–897

Fijan S, Honigsmann H and Ortel B (1995) Photodynamic therapy of epithelial skin tumours using Δ-aminolevulinic acid and desferrioxamine. *Br J Dermatol* **133**: 282–288

Fukuda H, Paredes S and Batlle AMC (1989) Tumour-localising properties of porphyrins. *In vitro* studies using the porphyrin precursor Δ-amino-levulinic acid, in free and liposome encapsulated forms. *Drug Des Deliv* **5**: 133–139

Fukuda H, Paredes S and Batlle AMC (1992*a*) Tumour-localising properties of porphyrins. *In vivo* studies using free and liposome encapsulated forms of Δ-aminolevulinic acid. *Comp Biochem Physiol* **102B**: 433–436

Fukuda H, Paredes S, Casas A, Chueke F and Batlle AMC (1992*b*) Potential of liposome-entrapped Δ-aminolevulinic acid in cancer therapy. Effect of prior injection of empty liposomes and different routes of administration. *Cancer J* **5**: 295–299

Fukuda H, Batlle AMC and Riley PA (1993) Kinetics of porphyrin accumulation in cultured epithelial cells exposed to ALA. *Int J Biochem* **25**: 1407–1410

Gardener LC, Smith SJ and Cox TM (1991) Biosynthesis of Δ-aminolevulinic acid and the regulation of heme formation by immature erythroid cells in man. *J Biol Chem* **266**: 22010–22018

Goldberg A and Rimington C (1962) *Diseases of Porphyrin Metabolism*. pp. 156–70. C Thomas:Springfield, Illinois, USA.

Granick S (1966) The induction of the synthesis of Δ-aminolevulinic acid synthase in chemical porphyria: a response to certain drugs, sex hormones and foreign chemicals. *J Biol Chem* **241**: 1359–1375

Granick S and Sassa S (1971) Delta-aminolevulinic acid synthase and the control of haem and chlorophyll synthesis. In *Metabolic Regulation*. Vogel HJ (ed), pp. 77–141. Academic Press: NY, USA

Granick S, Sinclair D, Sassa S and Grieninger G (1975) Effects by haem, insulin and serum albumin on haem and protein synthesis in chick embryo liver cells cultured in a chemically defined medium, and a spectrofluorometric assay for porphyrin composition. *J Biol Chem* **250**: 9215–9225

Hamilton JW, Bement WJ, Sinclair PR, Sinclair JF, Alcedo JA and Wetterhahn KE (1991) Heme regulates hepatic Δ-aminolevulate synthase mRNA expression by decreasing mRNA half-life and not by altering it rate of transcription. *Arch Biochem Biophys* **289**: 387–392

Iinuma S, Farshi SS, Ortel B and Hasan T (1994) A mechanistic study of cellular photodestruction with Δ-aminolevulinic acid-induced porphyrin. *Br J Cancer* **70**: 21–28

Kreigmair RM, Baumgartner R, Knuchel R, Stepp H, Hofstadter F and Hofstetter A (1996) Detection of early bladder cancer by Δ-aminolevulinic acid induced porphyrin fluorescence. *J Urol* **155**: 105–109

© Cancer Research Campaign 1997

Loh CS, Vernon D, MacRobert AJ, Bedwell J, Bown SG and Brown SB (1993) Endogenous porphyrin distribution induced by Δ-aminolevulinic acid in the tissue layers of the gastrointestinal tract. *J Photochem Photobiol B Biol* **20**: 47–54

Malik Z and Lugaci H (1987) Destruction of erythro-leukemic cells by photoactivation of endogenous porphyrins. *Br J Cancer* **156**: 589–595

May BK, Bhasker CR, Bawden MJ and Cox TC (1990) Molecular regulation of Δ-aminolevulinate synthase. Diseases related to heme biosynthesis. *Mol Biol Med* **7**: 405–421

Moretti MB, Garcia SRC, Stella C, Ramos EH and Batlle AMC (1993) Δ-aminolevulinic acid transport in *Saccharomyces cerevisiae*. *Int J Biochem* **25**: 1917–1924

Moretti MB, Garcia SRC, Chianelli MS, Ramos EH, Mattoon JR and Batlle AMC (1995) Evidence that γ-aminobutyric acid and Δ-aminolevulinic acid share a common transport system into *Saccharomyces cerevisiae*. *Int J Biochem Cell Biol* **27**: 169–173

Muller-Eberhard U and Nikkilä H (1989) Transport of tetrapyrroles by proteins. *Semin Hematol* **26**: 86–104

Rimington C (1989) Haem biosynthesis and porphyrias: 50 years in retrospect. *J Clin Chem Clin Biochem* **27**: 473–486

Roberts DJ and Cairnduff F (1995) Photodynamic therapy of primary skin cancer: a review. *Br J Plast Surg* **48**: 360–370

Sassa S and Kappas A (1977) Induction of Δ-aminolevulinate synthase and porphyrins in cultured liver cells maintained in chemically defined medium. Permissive effects of hormones on the induction process. *J Biol Chem* **252**: 2428–2436

Schwartz S, Dahl J, Ellefson M and Ahlquist D (1983) The 'Hemoquant' test: A specific and quantitative determination of heme (hemoglobin) in feces and other materials. *Clin Chem* **29**: 2061–2067

Smith PK, Krohn RI, Hermanson GT, Mallia AK, Gartner FH, Provenzano MD, Fujimoto EK, Goeke NM, Olson BJ and Klenk DC (1985) Measurement of protein using bicinchoninic acid. *Anal Biochem* **150**: 76–85

Steinbach P, Weingandt H, Baumgartner R, Kriegmair M, Hofstadter F and Knuchel R (1995) Cellular fluorescence of the endogenous photosensitizer protoporphyrin IX following exposure to Δ-aminolevulinic acid. *Photochem Photobiol* **6**: 887–895

© Cancer Research Campaign 1997